# Elongator and the role of its subcomplexes in human diseases

**DOI:** 10.15252/emmm.202216418

**Published:** 2022-11-30

**Authors:** Monika Gaik, Marija Kojic, Brandon J Wainwright, Sebastian Glatt

**Affiliations:** ^1^ Malopolska Centre of Biotechnology Jagiellonian University Krakow Poland; ^2^ Faculty of Medicine, The University of Queensland Diamantina Institute The University of Queensland Woolloongabba QLD Australia

**Keywords:** cancer, Elongator complex, Elp2, Elp4, Elp6, neurodevelopment, tRNA modification, Neuroscience, RNA Biology

## Abstract

The Elongator complex was initially identified in yeast, and a variety of distinct cellular functions have been assigned to the complex. In the last decade, several research groups focussed on dissecting its structure, tRNA modification activity and role in translation regulation. Recently, Elongator emerged as a crucial factor for various human diseases, and its involvement has triggered a strong interest in the complex from numerous clinical groups. The Elongator complex is highly conserved among eukaryotes, with all six subunits (Elp1‐6) contributing to its stability and function. Yet, recent studies have shown that the two subcomplexes, namely the catalytic Elp123 and accessory Elp456, may have distinct roles in the development of different neuronal subtypes. This Commentary aims to provide a brief overview and new perspectives for more systematic efforts to explore the functions of the Elongator in health and disease.

## Graphical abstract

This Commentary provides a brief overview and new perspectives for more systematic efforts to explore the functions of the Elongator in health and disease.

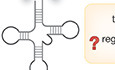

The dodecameric Elongator complex acetylates wobble uridines (U_34_) of 11 tRNA species (Abbassi *et al*, 2020), which is essential to keep optimal translation rates and maintain proteome integrity. Hypomodification of the Elongator‐dependent tRNAs profoundly impairs the ability of cells to balance protein homeostasis and results in protein misfolding and aggregation. Amino acid substitutions in almost all subunits of the complex have been associated with various neurodevelopmental and neurodegenerative disorders. The initial suspicion that an impaired function of Elongator directly causes the underlying neuropathology of the conditions was verified by studying conditional loss‐of‐function (LoF) murine models. Indeed, neuron‐specific LoF of the catalytic Elp3 subunit was found to impair tRNA modification and trigger the unfolded protein response (UPR) through interference with codon translation speed. As a consequence, murine Elp3 LoF models demonstrate defects in neurogenesis with associated microcephaly and neurodegeneration.

Familial dysautonomia (FD) is a well‐described Elongator‐related neurodevelopmental disorder. This rare disease is caused by a splice mutation in the ELP1/IKBKAP gene, resulting in a tissue‐specific deficiency in ELP1 protein, leading to impaired neuronal development and the survival of neurons. Understanding the genetic background of FD patients has facilitated the development of targeted therapies leading to prolonged survival and improved condition of affected patients (Morini *et al*, 2019). For instance, the neuronal defects of aberrantly spliced *ELP1* gene can be alleviated by mRNA splicing enhancers (Ohe *et al*, 2017) or proteasome inhibitors (Hervé & Ibrahim, 2017). The first pathogenic germline mutation in the mammalian Elongator complex predominantly affecting the CNS was identified in mice by an ENU mutagenesis screen on the basis of its wobbly gait (Kojic *et al*, 2018). The detected hypomorphic, missense mutation in the *Elp6* gene was shown to cause Purkinje neuron (PN) degeneration, resulting in cerebellar ataxia‐like phenotype in mice. Our groups have demonstrated that the mutation destabilized the complex and compromised its function in tRNA modification, leading to UPR and ER stress‐mediated apoptosis of PNs. The study provided the first mechanistic insight into the pathophysiology of cerebellar ataxia and neurodevelopment in general caused by an Elongator mutation.

Neurodegeneration in the *wobbly* mice was concomitant with substantial cerebellar neuroinflammation triggered by the NLRP3 inflammasome activation. Inhibition of microglial priming by blocking the inflammasome pathway both genetically and pharmacologically attenuated the PN degeneration and delayed the onset of ataxia in the *Elp6* mutants. Thus, the *wobbly* PNs not only die due to an intrinsic defect but are also killed by the excessive inflammatory response. Whether microgliosis is directly linked to the *Elp6* mutation or triggered by the neurodegeneration *per se* remains elusive. Yet, a recent study demonstrated that an Elongator mutation leads to an inflammatory condition (Chen *et al*, 2022). The study provides a direct link between impaired tRNA modification levels and tailored translational reprogramming during macrophage polarization. Elp3 deficiency promotes the pro‐inflammatory M1 state, leading to tissue damage and exacerbation of colitis in mice. The authors show that Elp3 limits M1 progression while promoting M2 macrophage polarization through activating translation of the mTORC2 regulator Ric8b in a codon‐dependent manner. Given the recent evidence that the NLRP3 pathway and Elongator itself are regulated by mTOR signalling in macrophages (Candiracci *et al*, 2019), there is a strong indication that the *Elp6* mutation may be directly responsible for active microgliosis in the *wobbly* mice.

## Mutations in different subunits lead to different neuronal phenotypes

Two functional studies modelling patient‐derived Elongator mutations in mice showed that proteotoxic stress‐induced PN loss was apparent with the mutations in either of the subcomplexes (Kojic *et al*, 2021; Gaik *et al*, 2022). The modelled biallelic point mutations in the *ELP2*, *ELP4* and *ELP6* genes originated from patients with intellectual disability, microcephaly, motor impairment, myelination defects and epilepsy. Patients with the *ELP2* mutations had additional autistic features. Modelling these variants in mice recapitulated the patient features. Brain imaging and tractography analysis of the *Elp2* mutants revealed microcephaly, loss of white matter tract integrity and an aberrant functional connectome. *In vitro* and *in vivo* studies demonstrated that the *ELP2*, *ELP4* and *ELP6* mutations impair the acetyl‐CoA activity of the complex and its function in translation via tRNA modification, resulting in perturbed protein homeostasis (Kojic *et al*, 2021; Gaik *et al*, 2022).

Although the common findings for the mutations in both subcomplexes were neurodevelopmental anomalies and PN degeneration in mice, the mutations surprisingly were found to affect different neuronal cells and structures. Microcephaly, cortical neurogenesis, myelination and interneuron defects observed in the *Elp2* mutants were not evident upon mutations in the accessory Elp456 subcomplex (Kojic *et al*, 2021; Gaik *et al*, 2022). Moreover, the Elp456 subcomplex seems to regulate the neurogenesis of the hippocampal pyramidal neurons, which is yet to be confirmed for the catalytic subcomplex. Biochemically, both subcomplexes appear as stand‐alone assemblies which interact with each other to effectively fulfil the crucial cycle of tRNA recognition, modification and release. Thus, the two subcomplexes seem to have distinct importance in brain development whereby Elp123 regulates the neurodevelopment and neuronal activity across a wide range of brain regions, and the hexameric Elp456 activity may be dispensable for the normal development of some of these structures. We can assume that mutations in the catalytic subcomplex affect all Elongator‐dependent tRNA species and thus, impact various neuronal subtypes, while *Elp456* mutations affect the binding of only a subset of Elongator‐dependent tRNAs. Indeed, analysing the affinity of the identified *Elp4*/*6* variants for *in vitro*‐transcribed tRNA species carrying Elongator‐modifiable U_34_ showed that the binding of tRNA^Ala^ was more affected than tRNA^Arg^, which was the first observation of tRNA specificity for the Elongator subcomplexes. Hence, only a subset of neuronal subtypes would be affected by the mutations in the Elp456 subcomplex, assuming that not all Elongator‐dependent tRNAs are expressed evenly in different neurons. This presumption is also supported by decreased level of tRNA modifications in patients' fibroblasts harbouring *ELP4*/*6* variants and by impeded activity of the Elongator complex *in vitro* (Gaik *et al*, 2022).

## Elongator mutations in cancer: drivers or tumour suppressors

A key role of the Elongator complex in tumour development and progression has been established for various cancer types. A number of studies have demonstrated that Elongator activity can promote cancer growth and metastasis by regulating malignant translational reprogramming of cells in skin, intestine, breast, bladder and hepatocellular carcinoma (Hawer *et al*, 2018). Mechanistically, Elp3‐dependent tRNA modifications promote glycolysis in melanoma cells via codon‐dependent regulation of HIF1A translation, which further enables the survival and therapy resistance of these tumours (Rapino *et al*, 2018). In breast cancer, Elp3 supports tumorigenesis through the translation of the oncoprotein DEK that promotes internal ribosomal entry site (IRES)‐dependent translation of the pro‐invasive transcription factor LEF1 (Delaunay *et al*, 2016). Elp3 ablation inhibited the invasion and metastasis of malignant cells in all of the above‐mentioned cancer types.

Recent studies have suggested an opposing role of the complex in tumorigenesis with LoF mutations in both subcomplexes initiating tumour development. Loss of ELP5 and ELP1 has been demonstrated to directly impede the wobble U_34_ tRNA modification leading to cancer of the gallbladder (Xu *et al*, 2019) and medulloblastoma (Waszak *et al*, 2020) respectively. The ELP5 LoF impairs the translation of hnRNPQ mRNA, a validated P53 IRES trans‐acting factor, leading to poor survival outcomes after chemotherapy treatment of gallbladder cancer (Xu *et al*, 2019). Tumours from patients with ELP1 LoF medulloblastoma were characterized by codon‐dependent translational reprogramming and induction of the UPR and loss of protein homeostasis (Waszak *et al*, 2020). Thus, Elongator mutations across different subunits lead to tRNA modification‐based translational defects in different types of cancer, affecting their growth and survival in different ways, which is likely dependent on the cancer type, its genomic landscape and microenvironment.

## Cell type specificity of Elongator and its tRNAs

**Figure 1:**
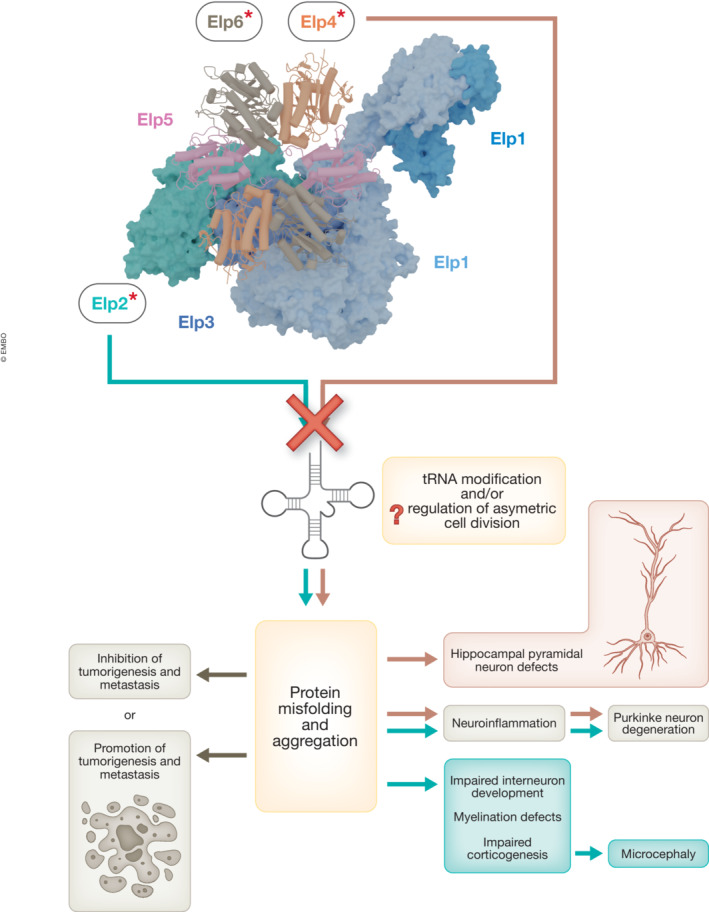
The effect of Elongator mutations in human diseases Model of the eukaryotic Elongator complex highlighting one of its Elp123 lobes (surface representation) and the bound Elp456 (cartoon representation). The six subunits (Elp1‐6) are labelled and subunits with known clinical mutations are marked with an asterisk. Different cellular consequences of an aberrant tRNA modification activity for tumorigenesis (left) and neurodegenerative disease (right) are illustrated below.

The plethora of recent studies have a clear common denominator. The Elongator complex is a crucial regulator of brain development, neuronal activity and carcinogenesis by managing ribosomal translation and maintaining proteome integrity (Fig 1). New lines of evidence point to different susceptibility of neuronal subtypes to tRNA modification defects induced by mutations in different subunits of the complex. Therefore, two key questions arose – (i) How different mutations in a common and ubiquitously expressed tRNA modification complex can lead to cell‐type‐specific phenotypes? (ii) Why do distinct neuronal subtypes show differential susceptibility to the variants in different elongator subunits? We speculate that tRNA selectivity, variable codon usage or the cell‐type‐specific expression of different tRNA iso‐acceptors could explain the observations. Further transcriptomic and epitranscriptomics studies are clearly needed to screen specific brain cells for the expression of distinct tRNA species in addition to their specialized proteomes. A greater understanding of the function of the complex in specific cancer types in synergy with other drivers/suppressors is necessary to elucidate its two‐faced nature in cancer prior to starting the search for effective ways to either block or sustain its function.

## Elongators function during cell division

Last but not least, a recent study describes a novel unexpected function of Elongator during asymmetric cell division sensory organ precursor (SOP) cells in *Drosophila* (Planelles‐Herrero *et al*, 2022). In detail, the authors showed that Elongator directly binds to the central spindle and drives the formation of an asymmetric central spindle by stabilizing microtubules differentially on the anterior side. Of note, this newly described moonlighting function is independent of the tRNA modification activity of Elongator, but still requires the fully assembled complete complex with both Elp123 and Elp456 subcomplexes. It remains to be shown whether this function is conserved in human neurons and/or neuronal progenitors that also undergo extensive asymmetric cell division. As translation is strongly limited during cell division, it is tempting to speculate that patient‐derived mutations that affect the integrity of the complex may compromise both functions, whereas mutations that affect tRNA modification would still preserve asymmetric cell division. Additional studies are required to understand if this novel function contributes to the observed neurodevelopmental defects and to the clinical manifestation of Elongator‐related diseases.

## Disclosure and competing interests statement

The authors declare that they have no conflict of interest.
